# Perioperative body weight change is associated with in-hospital mortality in cardiac surgical patients with postoperative acute kidney injury

**DOI:** 10.1371/journal.pone.0187280

**Published:** 2017-11-17

**Authors:** Chih-Chung Shiao, Ya-Ting Huang, Tai-Shuan Lai, Tao-Min Huang, Jian-Jhong Wang, Chun-Te Huang, Pei-Chen Wu, Che-Hsiung Wu, I-Jung Tsai, Li-Jung Tseng, Chih-Hsien Wang, Tzong-Shinn Chu, Kwan-Dun Wu, Vin-Cent Wu, for the National Taiwan University Hospital Study Group on Acute Renal Failure (NSARF)

**Affiliations:** 1 Division of Nephrology, Department of Internal Medicine, Saint Mary’s Hospital Luodong, Yilan, Taiwan, R.O.C.; 2 Saint Mary’s Junior College of Medicine, Nursing and Management, Yilan, Taiwan, R.O.C.; 3 Department of Nursing, Saint Mary’s Hospital Luodong, Yilan, Taiwan, R.O.C.; 4 Graduate Institute of Clinical Medical Sciences, Chang Gung University, Taoyuan, Taiwan, R.O.C.; 5 Division of Nephrology, Department of Internal Medicine, National Taiwan University Hospital, Taipei, Taiwan, R.O.C.; 6 Division of Nephrology, Department of Internal Medicine, Chi-Mei Medical Center, Liouying, Tainan, Taiwan, R.O.C.; 7 Division of Internal & Critical Care Medicine, Department of Critical care Medicine, Taichung Veterans General Hospital, Taichung, Taiwan, R.O.C.; 8 Division of Nephrology, Department of Internal Medicine, MacKay Memorial Hospital, Taipei, Taiwan, R.O.C.; 9 Division of Nephrology, Taipei Tzu Chi Hospital, Buddhist Tzu Chi Medical Foundation, Taipei, Taiwan, R.O.C.; 10 School of Medicine, Tzu Chi University, Hualien, Taiwan, R.O.C.; 11 Department of Pediatrics, National Taiwan University Hospital, Taipei, Taiwan, R.O.C.; 12 Department of Surgery, National Taiwan University Hospital, Taipei, Taiwan, R.O.C.; Kaohsiung Medical University Hospital, TAIWAN

## Abstract

**Background:**

Postoperative acute kidney injury (AKI) is common following cardiac surgery (CS). Body weight (BW) may be an amenable variable by representing the summation of the nutritional and the fluid status. However, the predictive role of perioperative BW changes in CS patients with severe postoperative AKI is never explored. This study aimed to evaluate this association.

**Methods:**

This study was conducted using a prospectively collected multicenter cohort, NSARF (National Taiwan University Hospital Study Group on Acute Renal Failure) database. The adult CS patients with postoperative AKI requiring renal replacement therapy (RRT), who had clear initial consciousness, received CS within 14 days of hospitalization, and underwent RRT within seven days after CS in intensive care units from January 2001 to January 2014 were enrolled. With the endpoint of 30-day postoperative mortality, we evaluated the association between the clinical factors denoting fluid status and patients outcomes.

**Results:**

A total of 188 patients (70 female, mean age 63.7 ± 15.2 years) were enrolled. Comparing with the survivors (n = 124), the non-survivors (n = 64) had a significantly higher perioperative BW change [3.6 ± 6.1% versus 0.1 ± 8.3%, p = 0.003] but not the postoperative and pre-RRT BW changes. By using multivariate Cox proportional hazards model, the independent indicators of 30-day postoperative mortality included perioperative BW change (p = 0.026) and packed red blood cells transfusion (p = 0.007), postoperative intra-aortic balloon pump (p = 0.001) and central venous pressure level (p = 0.005), as well as heart rate (p = 0.022), sequential organ failure assessment score (p < 0.001), logistic organ dysfunction score (p = 0.001), and blood total bilirubin level (p = 0.044) at RRT initiation. The generalized additive models further demonstrated, in a multivariate manner, that the mortality risk rose significantly during a perioperative BW change of 2% to 15%.

**Conclusions:**

Perioperative BW change was independently associated with an increased risk for 30-day postoperative mortality in CS patients with RRT-requiring AKI.

## Introduction

Acute kidney injury (AKI) is a common yet potentially serious complication after cardiac surgery (CS). Among the patients underwent CS, about 17–49% suffer from postoperative AKI, and 2–6% have severe AKI requiring renal replacement therapy (RRT). [1–3] The AKI has a dose-response relationship between its severity and the adverse effect on long-term survival of the patients. [4] The pathophysiology of postoperative AKI in CS patients is complex and multifactorial, which includes several factors such as exogenous and endogenous toxins, metabolic factors, ischemia-reperfusion injury, neurohormonal activation, embolization, hemodynamic alterations, along with inflammation and oxidative stress. These mechanisms likely interact with each other at different time points during the period from preoperative stage to postoperative stages of the CS. [5–7]

The postoperative prognoses after CS are varied with many factors such as patient characteristics, preexisting comorbidities, inflammatory activity, and duration of extracorporeal circulation. [8, 9] Besides, body weight (BW) or BW change is also an important but often overlooked indicator for patient outcomes in many clinical settings. [9, 10] It is accepted that an unintended BW loss of > 10% within six months is indicative of undernutrition and is associated with adverse patient outcomes. [11] In the patients undergoing CS, both preoperative unintended BW loss of ≧ 10% in the past 6 months and BMI ≦ 21.0 kg/m^2^ were found as independent indicators for a prolonged hospital length of stay (LOS) in a prospective cohort study by van Venrooij et al. [9] While a BW loss > 5% during the follow-up with a median period of 6 months was found as a significant risk factor for death and cardiovascular death in patients post myocardial infarction in a randomized clinical trial conducted by Lopez-Jimenez et al. [12] On the contrary, a BW gain ≧ 2 pounds (0.91 kg) over 3 days was a significant indicator for heart function decompensation and worse outcomes in patients with heart failure with preserved ejection fraction. [13] It is straightforward that the fluid retention secondary to the heart failure is majorly accountable for the increased BW.

Physiologically, the patient during the perioperative stage is in a stressful situation with highly dynamic and catabolic status which on the one hand would precipitate BW loss via the depletion of glycogen and subcutaneous fat, as well as the wasting of the muscle. [14] But on the other hand, the response to the stress of the patients may cause the BW gain by fluid retention, which is through the secretion of antidiuretic hormone, renin, and aldosterone. [15] In the aspect of fluid retention, excessive fluid accumulation is not only demonstrated to link with the raised morbidities and mortality in patients with critical illness or AKI, and those undergoing major surgeries, [16, 17] but also shown to be associated with higher risk of subsequent AKI. [18]

Among the CS patient with severe postoperative AKI, the RRT-requiring AKI worsens the patient outcomes, [19] which draws the attention at least on the issues of fluid status and nutrition. By representing the summation of the nutritional situation and the fluid status, BW may be an amenable variable as an intervention target in clinical practice. [10] However, the role of perioperative BW change in predicting patient outcomes in CS patients with RRT-requiring AKI is never explored previously. Thus, we conducted the current study aiming to evaluate this association in this population.

## Material and methods

### Study design and participants

This study was conducted using a prospectively collected multicenter cohort, NSARF (National Taiwan University Hospital Study Group on Acute Renal Failure) database [20–22]. The adult patients (aged > 18 years) who received CS with postoperative AKI requiring RRT support in intensive care units (ICUs) from January 2001 to January 2014 were enrolled. To purify the patient characteristics and limit the potential confounding factors, the inclusion criteria comprised the following: with clear consciousness at initial hospitalization, receiving CS within 14 days after hospitalization, [23] and undergoing RRT within seven days after the surgeries. [24]

The exclusion criteria included patients who were on chronic RRT or received acute RRT before the CS. The enrolled patients were categorized into two groups, survivors and non-survivors, based on the 30-day postoperative mortality. Then we evaluated the association between BW changes and patient survivals.

### Ethics, consent, and permissions

The Institutional Review Board of National Taiwan University Hospital approved the study (No. 31 MD03) and waived the need for informed consent because of the absence of both breach of privacy and interference with clinical decisions. The data were analyzed anonymously as well.

### Endpoint of current study

The endpoint of the current study is the 30-day postoperative mortality which was defined as patient mortality ≦ 30 days after surgeries. [25] The censoring period was calculated from the calendar day of receiving CS to the mortality (in non-survivors) or the 31st postoperative day (in survivors).

### Data collection

The baseline demographic data, comorbid diseases, Charlson Comorbidity Index (CCI), types of surgeries, etiology of AKI, modalities of RRT, and other remarkable information during the hospitalization were documented. This information included the amount of perioperative blood transfusion, diuretic therapy, as well as usage of intra-aortic balloon pump (IABP), extracorporeal membrane oxygenation (ECMO) support, and cardiopulmonary resuscitation (CPR).

Clinical parameters including hemodynamics and inotropic equivalents, [26] and laboratory tests including complete blood cell count, liver function, renal function, serum levels of relevant electrolytes, and arterial blood gas were recorded at the three-time points, namely, hospital admission, postoperative stage, and RRT initiation. The BW, presented with the unit of the kilogram, was measured every day by bed weighing scales which were calibrated.

The severity scores including the Glasgow Coma Scale (GCS), the Acute Physiology and Chronic Health Evaluation II (APACHE II) scores [27], the Multiple Organ Dysfunction Score (MODS), [28] the Logistic Organ Dysfunction Score (LODS), [29] and the Sequential Organ Failure Assessment (SOFA) scores [30], were also measured at the postoperative stage and RRT initiation. (S1 Appendix)

### Definitions

The perioperative BW change was defined as the percentage change of BW from “hospital admission” to “ICU admission immediately after CS (postoperative stage)” [**100% x (BW**_**ICU**_**−BW**_**hosp**_**)/ BW**_**hosp**_]. The postoperative change was the change from “ICU admission” to “RRT initiation” [**100% x (BW**_**ICU**_**−BW**_**hosp**_**)/ BW**_**hosp**_]. The pre-RRT change was the change from “hospital admission” to “RRT initiation” [**100% x (BW**_**RRT**_**−BW**_**hosp**_**)/ BW**_**hosp**_]. The post-RRT change was the change from “RRT initiation” to “hospital discharge or death” [**100% x (BW**_**dis**_**−BW**_**RRT**_**)/ BW**_**RRT**_]. Fluid overload was defined as the presence of both oliguria (urine output < 0.5ml/kg/hr for ≧12 hours) and severely impaired oxygenation (PaO2/FiO2 < 200). Clear consciousness was defined with GCS ≧14 points. Estimated glomerular filtration rate (eGFR) was calculated using the Chinese Modification of Diet in Renal Disease equation. [31]

Besides, the comorbidities including diabetes mellitus (DM), hypertension, chronic kidney disease (CKD), [32] sepsis, [33] shock, coronary artery disease (CAD), cerebrovascular accident (CVA), chronic obstructive pulmonary disease (COPD) and liver cirrhosis were defined according to previous medical records and/or relevant examinations. Moreover, heart failure was classified according to New York Heart Association functional classification.

The indications for RRT initiation included: (1) blood urea nitrogen (BUN) *>* 80 mg/dl and serum creatinine (sCr) *>* 2 mg/dl with uremic symptoms, (2) a central venous pressure level > 12 mmHg or pulmonary edema with a PaO2/FiO2 < 300, (3) serum potassium *>* 5.5 mmol/l refractory to treatment, (4) urine output < 100 ml/8 hours refractory to diuretics treatment, and (5) a pH *<* 7.2 in arterial blood gas.

The dialysis modality was chosen according to the hemodynamics of patients. Continuous venovenous hemofiltration (CVVH) was used if the dose of the inotropic equivalent of more than 15 points was required to maintain systolic blood pressure up to 120 mmHg. CVVH was performed with high-flux filters (Hemofilter, PAN-10, Asahi Kasei, Japan) using HF 400 (Infomed, Geneva, Switzerland) and a hemofiltration flow of 35 ml/kg per hour with a blood flow of 200 ml per minute. Replacement fluid was bicarbonate-buffered and was administered predilutionally at a dynamically adjusted rate to achieve the desired fluid therapy goals. Default composition was 142 mEq/l Na, 33 mEq/l bicarbonate, 1.4 mEq/l Mg, and 2.6 mEq/l Ca. Hemodialysis was performed using low-flux polysulfone hemofilter (KF-18C, Kawasumi Laboratories, Japan). Conventional intermittent hemodialysis was performed for four hours except for the first and second sessions with a blood flow of 200 ml/min and a dialysate flow of 500 ml/min. Hemodialysis adequacy assessment was measured: KT/V = [(in vitro urea clearance)× (prescribed time)]/predialysis total body water. The urea distribution volume is roughly equal to the total body water. Vascular access was obtained by percutaneous placement of a double-lumen catheter.

### Statistical analysis

The statistical analyses were performed using the Scientific Package for Social Science (PASW Statistics for Windows, Version 22.0, Chicago: SPSS Inc) and the *R* 2.12.1 (R Foundation for Statistical Computing, Vienna, Austria) software. A variable was deleted if the missing data > 5% or was replaced by linear interpolation if the missing data < 5%.

Categorical variables were expressed as “numbers (percentages).” Continuous variables were expressed as “mean ± standard deviation (SD)” for normal distribution and “mean ± SD [median, interquartile range (IQR)]” for non-normal distribution. The chi-square test, or the Fisher’s exact test if the expected value of any box is ≦ 5, was used to analyze categorical variables. The independent t-test for normal distribution or Mann-Whitney U test for non-normal distribution was used to compare continuous variables.

If the perioperative or the pre-RRT BW change was demonstrated to have a significant association with mortality in the independent t-test, the generalized additive model (GAM) was applied to present the change of death risk according to the percentage BW changes and to further determine the cut-point for postoperative mortality. The Kaplan-Meier survival curves with log-rank test were drawn to compare the patient survival between groups categorized by the cut-point determined by GAM.

Subsequently, the clinical variables at patients’ initial admission, perioperative stage, postoperative stage and pre-RRT stage were considered as candidate variables for Cox proportional hazards model. The multivariate Cox proportional hazards model with stepwise selection method was used to evaluate the independent indicators of the 30-day mortality from the variables which showed significances (p ≦ 0.05) in univariate analysis, and to investigate their regression coefficient, hazard ratio (HR), 95% confidence interval (CI) and p-value.

Finally, the receiver operating characteristic (ROC) curve with an area under the curve (AUC), along with the sensitivity, specificity, positive predictive value (PPV), and negative predictive value (NPV) was used to exam the predictive ability for postoperative mortality of the multivariate Cox proportional hazards model. Besides, the GAM was applied again to measure the probability of the death and draw the relationship plots between postoperative mortality and BW changes in the perioperative and postoperative period. In all statistical analyses, a two-sided p≦ 0.05 was considered statistically significant.

## Results

**Fig 1:**
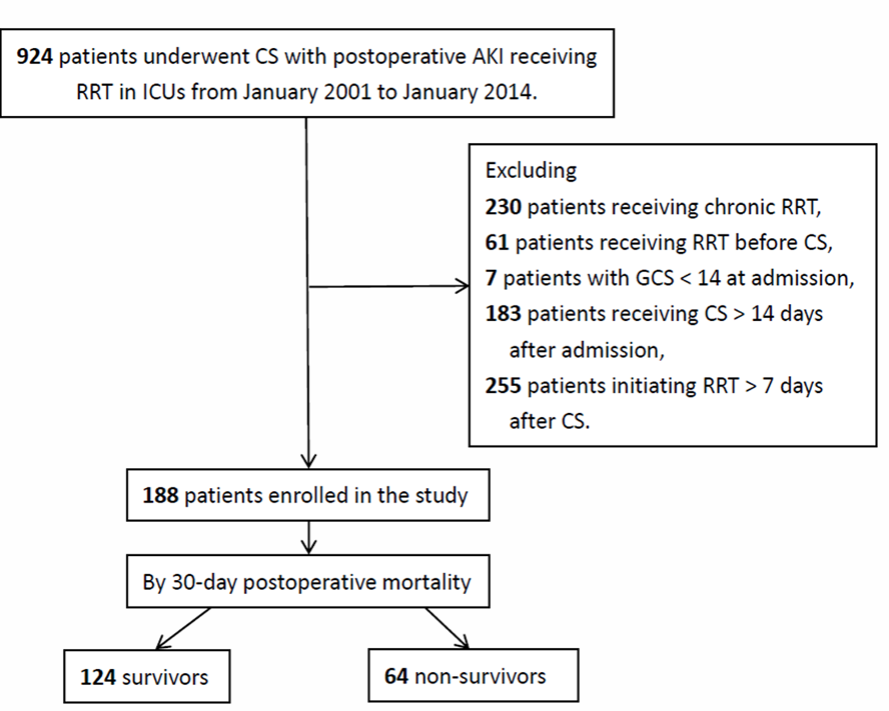
Flow chart of patient enrollment. **Abbreviations:** CS, Cardiac surgery; ICU, Intensive Care Unit; GCS, Glasgow Coma Scale; RRT, Renal Replacement Treatment.

During the enrollment period from January 2001 to January 2014, a total of 924 patients underwent CS and RRT in the ICUs. After excluding 230 patients who undergoing chronic RRT, 61 patients whose RRT were undertaken before the CS, 7 patients with GCS < 14 points at hospital admission, 183 patients received CS > 14 days after hospital admission, and 255 patients received RRT > 7 days after CS, 188 patients (70 female, mean age 63.7 ± 15.2 years) were enrolled. The enrollees were categorized into survivors (n = 124, 66.0%) and non-survivors (n = 64, 34.0%). (Fig 1) In non-survivors, the median duration from CS to death was ten days. With the setting of α 0.05 and odds ratio (OR) 2.145, the estimated power of logistic regression model in the current study was calculated as 0.98 by using the G-Power. [34].

### Clinical characteristics and variables

Compared to the survivors, the non-survivors had a higher proportion of having DM, but a lower proportion of CKD (p < 0.001) and lower values of CCI. The non-survivors received fewer units of perioperative packed red blood cells (PRBC) transfusion but had a higher probability of using IABP. They also had a higher probability of a shock as the etiology of AKI (p = 0.002) and used CAVH as the majority of RRT modality (p < 0.001). (all p = 0.001 except otherwise expressed) (Table 1)

**Table 1:** Comparisons of basic characteristics and important clinical variables between survivors and non-survivors.

Variable	Survivors (n = 124)	Non- Survivors (n = 64)	p*-*value
***Basic demography***			
Gender, female	46 (37.1%)	24 (37.5%)	0.957
Age, years	62.7 ± 15.3 [65.8, 77.0]	65.7 ± 15.0 [69.0, 64.0]	0.145*
BMI, kg/m^2^	23.7 ± 3.8	23.0 ± 3.5	0.250
Smoking	19 (15.3%)	5 (7.8%)	0.144
Comorbid disease			
CAD	37 (29.8%)	14 (21.9%)	0.245
DM	69 (55.6%)	51 (79.7%)	0.001
Hypertension	76 (61.3%)	37 (57.8%)	0.644
Heart failure			0.264
NYHA Fc-I	44 (35.5%)	18 (28.1%)	
NYHA Fc-II	45 (36.3%)	33 (51.6%)	
NYHA Fc-III	22 (17.7%)	8 (12.5%)	
NYHA Fc-IV	2 (1.6%)	2 (3.1%)	
CKD	25 (20.2%)	1 (1.6%)	< 0.001
Severe COPD	3 (2.4%)	1 (1.6%)	0.700
CVA	5 (4.0%)	0 (0.0%)	0.103
Cirrhosis of liver	3 (2.4%)	1 (1.6%)	0.700
Charlson Comorbidity Index	2.79 ± 2.87 [2, 12]	1.52 ± 0.69 [2, 4]	0.100*
**Categories of surgeries**			0.975
Valvular replacement	44 (35.5%)	22 (34.4%)	
CABG	43 (34.7%)	26 (40.6%)	
CABG+valvular replacement	9 (7.3%)	4 (6.4%)	
Heart transplantation	3 (2.4%)	1 (1.6%)	
Endovascular stent grafting	9 (7.3%)	5 (7.8%)	
Others	16 (12.9%)	6 (9.4%)	
**Perioperative blood transfusion**			
Whole Blood, IU	0.16 ± 0.75 [0, 5]	0.21 ± 0.79 [0, 4]	0.426*
Packed red blood cells, IU	1.57 ± 3.92 [0, 33]	0.30 ± 1.11 [0, 7]	0.002*
Fresh frozen plasma, IU	2.77 ± 3.60 [3, 30]	3.73 ± 3.44 [3, 18]	0.017*
Platelet, IU	6.66 ± 7.30 [6, 60]	8.34 ± 8.24 [6, 42]	0.188*
Cryoprecipitate, IU	1.01 ± 3.87 [0, 24]	0.72 ± 2.05 [0, 12]	0.238*
**Etiology of AKI**			0.957
Shock	104 (83.9%)	57 (89.1%)	0.002
Sepsis	27 (21.8%)	10 (15.6%)	0.957
**Modality of RRT**			<0.001
IHD	29 (23.4%)	0 (0.0%)	
CVVH	5 (4.0%)	0 (0.0%)	
CAVH (with ECMO)	47 (37.9%)	57 (89.1%)	
Mixed method	22 (17.7%)	5 (7.8%)	
SLEDD-f / SLEDD	21 (16.9%)	2 (3.1%)	
**Other important remarks**			
Intra-aortic balloon pump	10 (8.1%)	16 (25.0%)	0.001
Cardiopulmonary resuscitation	6 (4.8%)	0 (0.0%)	0.074
Post-op diuretics use	78 (62.9%)	48 (75.0%)	0.095
Duration of CS, hours	4.5±2.0 [4.2, 13.3]	5.1±2.2 [4.5, 12.4]	0.084*
Hospital to ICU, day	4.5 ± 3.6 [3, 13]	4.2 ± 3.5 [3, 13]	0.715*
ICU to RRT, day	1.9 ± 1.4 [1, 6]	1.9 ± 1.4 [1, 5]	0.501*
Hospital to RRT, day	6.3 ± 4.0 [4, 5]	6.0 ± 3.8 [4, 5]	0.681*
Peri-op BW change, %	0.1 ± 8.3 [0.0, 65.0]	3.6 ± 6.1 [0.6, 24.2]	0.012*
Post-op BW change, %	4.2 ± 11.4 [2.3, 44.4]	2.7 ± 6.0 [4.0, 38.2]	0.335*
Pre-RRT BW change, %	3.6 ± 6.9 [0.0, 104.8]	6.4 ± 8.3 [0.4, 29.7]	0.841*
Post-RRT BW change, %	-10.2 ±14.6 [-8.3, 67.7]	0.7 ±10.2 [0.4, 52.3]	<0.001*
Fluid overload at ICU (post-op stage)^##^	44 (33.1%)	13 (20.3%)	0.067
Fluid overload at RRT initiation^##^	32 (25.8%)	21 (32.8%)	0.312

**Note:** Categorical variables were expressed as numbers (percentages) and analyzed using Chi-square test, or Fisher’s exact test if the expected value of any box is ≦5.

Continuous variables with normal distribution were expressed as “mean ± standard deviation” and analyzed using independent t-test.

*Continuous variables with non-normal distribution were expressed as “mean ± standard deviation [median, interquartile range]” and compared using Mann-Whitney U test.

^##^ Fluid overload was defined as the presence of both oliguria (urine output <0.5ml/kg/h for ≧12 hrs) and severely impaired oxygenation (PaO2/FiO2<200).

**Abbreviations:** AKI, acute kidney injury; BMI, body mass index; CABG, coronary artery bypass grafting; CAD, coronary artery disease; CAVH, continuous arteriovenous hemofiltration; CKD, chronic kidney disease; COPD, chronic obstructive pulmonary disease; CS, cardiac surgery; CVA, cerebrovascular accident; CS, cardiac surgery; CVVH, continuous venovenous hemofiltration; DM, diabetes mellitus; ECMO, extracorporeal membrane oxygenation; ICU, intensive care unit; IHD, intermittent hemodialysis; NYHA Fc, New York Heart Association functional classification; Peri-op, perioperative; Post-op, postoperative; PRBC, packed red blood cells; RRT, renal replacement therapy; SLEDD, slow extended daily dialysis; SLEDD-f, sustained low-efficiency daily diafiltration

Besides, the non-survivors had higher eGFR from hospital admission (p = 0.002) to the postoperative stage (p = 0.002) and at RRT initiation (p = 0.001). They also had higher hemoglobin at hospital admission. At both postoperative stage and RRT initiation, the non-survivors had lower blood pressure (BP), serum albumin, and GCS, as well as higher values of some electrolytes and liver biochemistries, higher heart rate (HR) and higher severity scores (the SOFA and the LODS at postoperative stage, along with the APACHE-II, the SOFA, the LODS, and the MODS at RRT initiation). As to the central venous pressure (CVP) levels, the non-survivors had lower CVP levels at postoperative stage (p = 0.032) but higher at RRT initiation (p = 0.013) comparing with the survivors. (Table 2 and S1–S3 Tables)

**Table 2:** Laboratory and clinical variables with statistical differences between survivors and non-survivors.

Variable	Survivors	Non- Survivors	p-value
	(n = 124)	(n = 64)	
**At hospital admission**			
***Laboratory tests***			
Hemoglobin, g/dL	11.4 ± 2.5 [11.2, 14.5]	12.2 ± 2.6 [12.2, 12.5]	0.020*
Hematocrit, %	34.6 ± 7.6 [33.4, 44.1]	37.1 ± 7.6 [36.3, 35.4]	0.023*
BUN, mg/dL	46.7 ± 29.5 [41.2, 118.7]	34.4 ± 34.4 [27.2, 89.7]	0.010*
Creatinine, mg/dL	3.4 ± 6.1 [2.1, 65.4]	1.9 ± 1.4 [1.4, 7.4]	0.002*
eGFR, ml/min/1.73m^2^	39.5 ± 29.5 [33.4, 145.6]	51.5 ± 27.7 [50.2, 130.4]	0.002*
GOT, IU/L	56.9 ± 120.8 [26.0, 1153.0]	155.9 ± 665.5 [34.0, 5318.0]	0.002*
Bil-T, mg/dL	1.1 ± 1.1 [0.7, 8.1]	1.4 ± 1.3 [1.0, 7.2]	0.008*
**At ICU admission (postoperative stage)**			
***Hemodynamics and vital signs***			
HR, /min	98.5 ± 21.2	106.6 ± 20.1	0.012
SBP, mmHg	125.2 ± 32.2	108.7 ± 26.6	0.001
DBP, mmHg	63.9 ± 16.5	58.5 ± 15.6	0.030
MAP, mmHg	84.3 ± 19.6	75.2 ± 16.9	0.002
CVP, mmHg	12.0 ± 5.3	10.4 ± 4.9	0.039
***Laboratory tests***			
WBC, 10^3^/uL	11.7± 4.8 [11.4, 26.0]	10.5 ± 5.8[1.0, 0.3]	0.020*
BUN, mg/dL	40.4 ± 21.4 [36.4, 118.2]	33.3 ± 21.2 [28.1, 109.9]	0.007*
sCr, mg/dL	3.3 ± 5.1 [2.4, 55.6]	2.1 ± 1.3 [1.7, 7.2]	0.003*
eGFR, ml/min/1.73m^2^	32.9 ± 24.6 [26.1, 146.1]	39.4 ± 16.1 [41.2, 101.6]	0.002*
Albumin, g/dL	3.4 ± 0.6	3.0 ± 0.7	0.004
Sodium, mEq/L	139.0 ±7.3 [139.0, 32.0]	144.2 ± 7.8 [143.0, 48.0]	<0.001*
GOT, IU/L	120.5 ± 155.7 [66.5, 1210.0]	318.2 ± 442.0 [167.5, 2084.0]	<0.001*
Bil-T, mg/dL	2.9 ± 9.4 [1.3, 102.0]	2.9 ± 2.6 [2.0, 14.0]	<0.001*
Lactate, mEq/L	6.4 ± 5.7 [4.7, 24.6]	9.1 ± 6.5 [6.95, 23.8]	0.002*
PaO2, mmHg	140.8 ± 79.8 [113.5, 427.5]	167.5 ± 87.0 [133.4, 346.3]	0.017*
***Severity scores***			
GCS, points	13.1 ± 4.1 [15, 12]	10.3 ± 5.2 [15, 12]	<0.001*
SOFA Score, points	9.0 ± 3.2 [9, 15]	11.1 ± 3.2 [11, 15]	<0.001*
LODS, points	14.2 ± 3.4 [15, 12]	16.5 ± 1.2 [17, 5]	<0.001*
**At RRT initiation**			
***Hemodynamics and vital signs***			
HR, /min	99.1 ± 20.2	109.1 ± 21.1	0.002
SBP, mmHg	123.5 ± 27.4	105.8 ± 24.9	<0.001
DBP, mmHg	63.1 ± 13.9	57.5 ± 15.1	0.011
MAP, mmHg	83.3 ± 16.2	73.6 ± 16.2	<0.001
CVP, mmHg	12.3 ± 5.1 [12, 25]	14.1 ± 5.4 [13, 31]	0.013*
IE, mcg/kg/min	34.8 ± 243.5 [6.7, 2719.4]	25.2 ± 24.3 [19.1, 168.2]	<0.001*
***Laboratory tests***			
Platelet, 10^3^/uL	133.3 ± 62.3 [129.5, 344.0]	108.4 ± 64.6 [94.5, 350.0]	0.004*
BUN, mg/dL	54.0 ± 29.0 [49.9, 156.7]	45.1 ± 24.8 [36.9, 99.4]	0.029*
sCr, mg/dL	4.3 ± 6.7 [3.2, 73.4]	2.7 ± 1.6 [2.1, 7.0]	<0.001*
eGFR, ml/min/1.73m^2^	24.0 ± 17.3 [19.3, 90.8]	30.4 ± 14.6 [29.6, 61.0]	0.001*
Albumin, g/dL	3.3 ± 0.6	3.0 ± 0.7	0.017
Sodium, mEq/L	140.3 ± 6.6 [139.3, 36.2]	145.9 ± 7.5 [145.0, 37.0]	<0.001*
Calcium, mg/dL	1.2 ± 0.1 [1.2, 0.9]	1.1 ± 0.1 [1.1, 0.7]	0.006*
GOT, IU/L	208.9 ± 516.6 [69.0, 5067.0]	649.3 ± 1706.8 [223.0, 12999.0]	<0.001*
Bil-T, mg/dL	2.4 ± 3.2 [1.3, 23.6]	3.8 ± 4.4 [2.3, 30.2]	<0.001*
Lactate, mEq/L	5.4 ± 5.4 [3.2, 26.5]	8.6 ± 6.4 [5.9, 26.1]	<0.001*
***Severity scores***			
GCS, points	13.4 ± 3.7 [15, 12]	9.4 ± 5.1 [8, 12]	<0.001*
APACHE-II, points	10.7 ± 5.8 [9.5, 31.0]	14.6 ± 7.3 [16, 29.0]	<0.001*
SOFA Score, points	10.2 ± 3.1 [10.0, 13.0]	13.9 ± 3.4 [14.0, 14.0]	<0.001*
LODS, points	13.6 ± 3.5 [15.0, 13.0]	15.8 ± 1.7 [17.0, 5.0]	<0.001*
MODS, points	7.1 ± 3.0 [6.5, 16.0]	10.0 ± 3.6 [9.5, 17.0]	<0.001*

**Note:** Categorical variables were expressed as numbers (percentages) and analyzed using Chi-square test, or Fisher’s exact test if the expected value of any box is ≦5.

Continuous variables with normal distribution were expressed as “mean ± standard deviation” and analyzed using independent t-test.

*Continuous variables with non-normal distribution were expressed as “mean ± standard deviation [median, interquartile range]” and compared using Mann-Whitney U test.

**Abbreviations:** APACHE, Acute Physiology and Chronic Health Evaluation; Bil-T, total bilirubin; BUN, blood urea nitrogen; CVP, central venous pressure; DBP, diastolic blood pressure; eGFR, estimated glomerular filtration rate; GCS, Glasgow Coma Scale; GOT, glutamate oxaloacetate transaminase; HR, heart rate; IE, inotropic equivalent; LODS, Logistic Organ Dysfunction Score; MAP, mean arterial pressure; MODS, Multiple Organ Dysfunction Score; PaO2, partial arterial pressure of oxygen; SBP, systolic blood pressure; sCr, serum creatinine; SOFA, Sequential Organ Failure Assessment; WBC, white blood cell.

### Body weight change and postoperative outcome

Compared to the survivors, the non-survivors had significantly higher perioperative BW change (3.6 ± 6.1% versus 0.1 ± 8.3%, p = 0.012) and pre-RRT BW change (6.4 ± 8.3 versus 3.6 ± 6.9, p = 0.841). An increase of 1% perioperative BW change was associated with 0.07 folds increase in the 30-day postoperative mortality after adjustment.

However, there were no significant differences between non-survivors and survivors in the postoperative BW change (2.7 ± 6.0% versus 4.2 ± 11.4%, p = 0.335), as well as the proportion of presenting fluid overload at postoperative stage (20.3% versus 33.1%, p = 0.067) and at RRT initiation (32.8% versus 25.8%, p = 0.312). Meanwhile, the period from hospital admission to postoperative stage (4.2 ± 3.5 versus 4.5 ± 3.6 days, p = 0.715), the period from postoperative stage to RRT initiation (1.9 ± 1.4 versus 1.9 ± 1.4 days, p = 0.501), and the period from hospital admission to RRT initiation (6.0 ± 3.8 versus 6.3 ± 4.0 days, p = 0.681) were also not statistically different between the non-survivors and the survivors. (Table 1)

**Fig 2:**
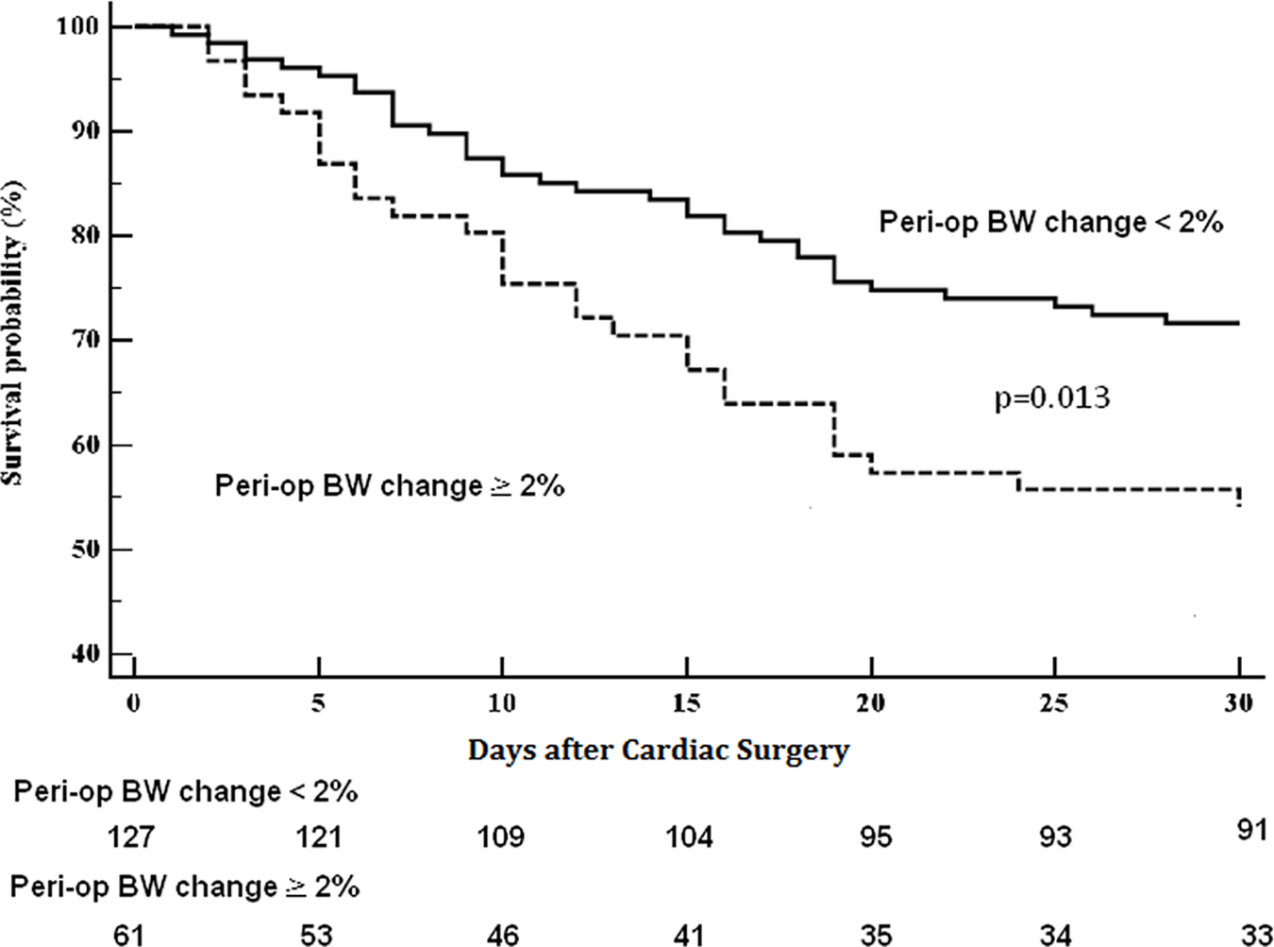
Kaplan-Meier survival curves of the two groups categorized by the perioperative body weight change of 2%. **Note:** The survival period was calculated from the day of receiving cardiac surgeries. Solid line and dashed line denoted the patients with perioperative BW change < 2% (n = 217) and 2% (n = 61), respectively. The 30-day postoperative mortality was significantly higher in the group with perioperative BW change≧ 2% (45.9%) than those with perioperative BW change < 2% (28.4%) (p = 0.013). **Abbreviations:** BW, body weight; Peri-op, perioperative.

By using the GAM, the estimated probability of death at 30-day started to increase significantly when the perioperative BW change reached a cut-point of 2.0%. (plot not shown) Moreover, we found that the patients with perioperative BW change ≧ 2.0% had significantly higher 30-day postoperative mortality than those with perioperative BW change < 2.0% (mortality rate, 45.9% versus 28.4%, p = 0.013). (Fig 1) Otherwise, the hospital LOS [45.0 ± 53.2 versus 47.5 ± 44.6 days, p = 0.743], the ICU LOS (17.3 ± 23.1 versus 17.2 ± 18.3 days, p = 0.989), the duration of ventilator support (22.8 ± 49.2 versus 16.5 ± 17.9 days, p = 0.208), and the duration from the CS to RRT initiation (1.6 ± 1.2 versus 2.0 ± 1.5 days, p = 0.064) were noted of significant differences between the groups with perioperative BW change ≧ 2% and < 2%. (Fig 2)

### Predictors of 30-day postoperative mortality

Then we evaluated the influences of all the variables including basic characteristics and clinical variables using the univariate Cox proportional hazards model. The variables with p≦ 0.05 on univariate analysis were put into the multivariate model for further analysis. These selected variables for multivariate analysis included DM, CKD, and some laboratory tests [hemoglobin, eGFR, GOT, and bilirubin (Bil-T)] at hospital admission; the clinical variables [HR, mean arterial pressure (MAP), CVP, IABP, perioperative PRBC and FFP transfusion, perioperative BW change and BW change after RRT], the laboratory tests (WBC, eGFR, sodium, glutamic oxaloacetic transaminase (GOT), Bil-T, albumin, lactate, PaO2), and the severity scores (GCS, SOFA Score and LODS) at postoperative stage; as well as the clinical variables (shock as etiology of AKI, modality with CAVH of RRT, HR, MAP, CVP, IE score), the laboratory tests (platelet, eGFR, sodium, calcium, GOT, albumin, Bil-T, lactate) and the severity scores (GCS, APACHE-II, SOFA Score, LODS and MODS) at RRT initiation. Also, a propensity score composed of age, body mass index, gender, CCI, eGFR, as well as the days between hospital admission to surgery, was applied for the baseline difference adjustment in the multivariate Cox proportional hazards model.

The multivariate Cox proportional hazards model subsequently disclosed several independent predictors of 30-day postoperative mortality. These independent predictors included perioperative BW change (HR 1.07, 95% CI 1.03–1.12, p = 0.001), Post-RRT BW change (HR 1.06, 95% CI 1.04–1.08, p <0.001), perioperative PRBC transfusion (HR 0.65, 95% CI 0.51–0.82, p <0.001), Modality with CAVH (HR 4.09, 95% CI 1.85–9.05, p = 0.001), HR at RRT (HR 1.02, 95% CI 1.01–1.03, p = 0.001), SOFA score at RRT (HR 1.22, 95% CI 1.13–1.33, p<0.001). (Table 3 and S4 Table) The predictive ability for the postoperative mortality of the perioperative BW change persisted when taking the situation of fluid overload at the postoperative stage and RRT initiation for adjustment. (HR 1.045, 95% CI 1.004–1.088, p = 0.032; and HR 1.046, 95% CI 1.006–1.088, p = 0.023, respectively).

**Table 3:** Independent predictors of 30-day postoperative mortality using multivariate Cox proportional hazards model.

	B	SE	HR	95% CI	p-value
Peri-op BW change ^a^	0.07	0.02	1.07	1.03–1.12	0.001
Post-RRT BW change ^a^	0.06	0.01	1.06	1.04–1.08	<0.001
Peri-op PRBC transfusion ^a^	-0.44	0.12	0.65	0.51–0.82	<0.001
CAVH (with ECMO) ^b^	1.41	0.41	4.09	1.85–9.05	0.001
HR at RRT ^a^	0.02	0.01	1.02	1.01–1.03	0.001
MAP at RRT^a^	-0.02	0.01	0.98	0.97–1.00	0.024
SOFA Score at RRT ^a^	0.20	0.04	1.22	1.13–1.33	<0.001

**Notes:** Variables were selected to put into multivariate analysis if they had a p ≦ 0.05 on univariate analysis. These selected variables for multivariate analysis included DM, CKD, and some laboratory tests (hemoglobin, eGFR, GOT, and Bil-T) at hospital admission; the clinical variables (HR, MAP, CVP, IABP, perioperative PRBC and FFP transfusion, perioperative BW change and BW change after RRT), the laboratory tests (WBC, eGFR, sodium, GOT, Bil-T, albumin, lactate, PaO2), and the severity scores (GCS, SOFA Score and LODS) at postoperative stage; as well as the clinical variables [shock as etiology of AKI, CAVH (with ECMO), HR, MAP, CVP, IE], the laboratory tests (platelet, eGFR, sodium, calcium, GOT, albumin, Bil-T, lactate) and the severity scores (GCS, APACHE-II, SOFA Score, LODS and MODS) at RRT initiation. Also, a propensity score composed of age, body mass index, gender, CCI, eGFR, as well as the days between hospital admission to surgery, was applied for the baseline difference adjustment in the multivariate Cox proportional hazards model.

Duration for analysis is measured using calendar days from cardiac surgery to mortality.

^a^ every increment of 1 unit or point

^b^ with versus without; “at RRT” denotes “at the timing of RRT initiation.”

**Abbreviations:** APACHE, Acute Physiology and Chronic Health Evaluation; B, beta coefficient; Bil-T, total bilirubin; BW, body weight; CAVH, continuous arteriovenous hemofiltration; CCI, Charlson Comorbidity Index; CI, confidence interval; CKD, chronic kidney disease; CVP, central venous pressure; DM, diabetes mellitus; ECMO, extracorporeal membrane oxygenation; eGFR, estimated glomerular filtration rate; FFP, fresh frozen plasma; GCS, Glasgow Coma Scale; GOT, glutamate oxaloacetate transaminase; HR, heart rate; HR, hazard ratio; IABP, intra-aortic balloon pump; IE, inotropic equivalent; LODS, Logistic Organ Dysfunction Score; MAP, mean arterial pressure; MODS, Multiple Organ Dysfunction Score; PaO2, partial arterial pressure of oxygen; Peri-op, perioperative; Post-op, postoperative; PRBC, packed red blood cell; RRT, renal replacement therapy; SE, standard error; SOFA, Sequential Organ Failure Assessment; WBC, white blood cell

**Fig 3:**
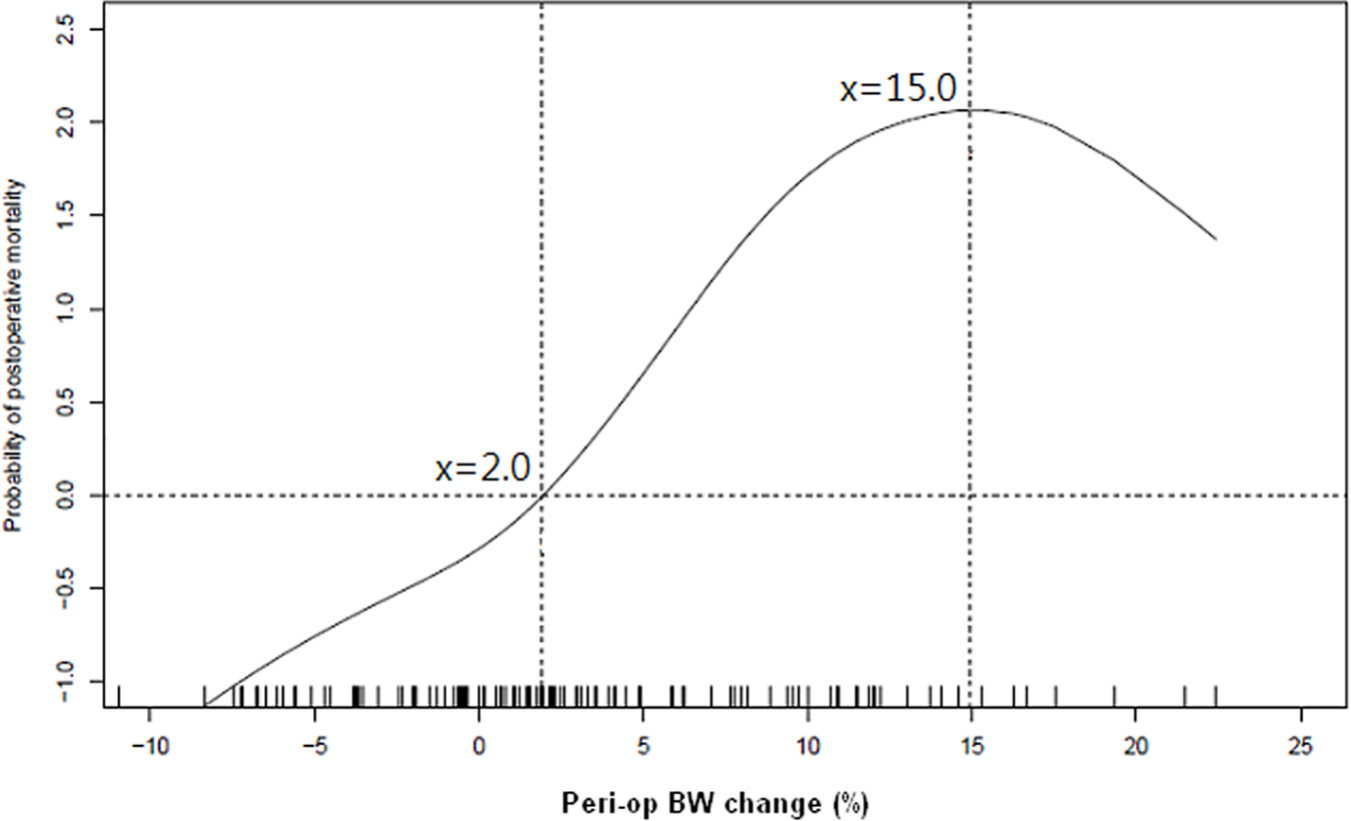
The probability of 30-day postoperative mortality according to the perioperative body weight changes. **Note:** The plot was drawn using a generalized additive model with adjustment for all other independent predictors, including perioperative packed red blood cells transfusion, postoperative intra-aortic balloon pump use, postoperative central venous pressure level, as well as heart rate, Sequential Organ Failure Assessment Score, Logistic Organ Dysfunction Score, and blood total bilirubin level at renal replacement therapy initiation. The estimated probability of 30-day postoperative mortality raised gradually accompanying the increase of perioperative BW change. The estimated probability started to increase significantly when the perioperative BW change reached 2.0% and reached the peak level when the BW change arrived at 15.0%. **Abbreviations:** BW, body weight; Peri-op, perioperative.

From the plot drawn using the GAM with adjustment to all other independent predictors mentioned above, we found that the probability of postoperative mortality significantly raised when perioperative BW change arrived 2.0% and progressed until the peak at perioperative BW change of 15.0%. (Fig 3)

Finally, the predictability of the current multivariate Cox proportional hazards model for postoperative mortality was proved to be excellent by the ROC analysis. (AUC 0.925, 95% CI 0.877–0.958, p<0.001, sensitivity 0.94; specificity 0.77, PPV 0.67, NPV 0.96).

## Discussions

Since CS-associated AKI is different from septic AKI in pathophysiologic mechanisms and patient prognoses, we used relatively strict inclusion criteria to minimize the influence of other confounding factors such as sepsis and to conduct a “relatively pure” cohort of CS patients with severe postoperative AKI. In a recently published single-center matched cohort study evaluating long-term outcomes for CS patients with dialysis-requiring AKI, the independent risk factors associated with elevated all-cause mortality were demonstrated to be increased age, with history of congestive heart failure, lower preoperative SCr, longer interval between CS and RRT initiation, and the need for mechanical ventilation or an intra-aortic balloon pump at RRT initiation. [19] Because of the different “time limits from CS to RRT initiation” in the inclusion criteria of the study by Thongprayoon et al. [19] (≦ 30 days) and the current study (≦ seven days), the study populations were different between the two studies. Furthermore, Thongprayoon et al. [19] did not take the variables about BW changes into consideration as risk factors.

To the best of our knowledge, the current prospective multicenter cohort study is the first investigation to evaluate the association between perioperative BW change and postoperative 30-day mortality in CS patients with RRT-requiring postoperative AKI. The 30-day mortality rate of CS patients with severe postoperative AKI requiring RRT in our study was 34.0%, which was comparable with the 32.5% of the CS patients with AKI (including mild form AKI) in the previous study. [35]

### Body weight change and patient outcomes

In the selected population, the risk of postoperative mortality significantly raised when the perioperative BW change arrived 2.0%. A BW gain of ≧ 2% carries a twofold probability of 30-day mortality than those whose BW gain < 2%. Even after adjustment for baseline differences using propensity score, and with fluid overload at either postoperative stage or RRT initiation, the perioperative BW change in continuous form was proved as an independent indicator for 30-day mortality.

BW change is a useful marker for fluid storage outside the circulatory space, [36] and is a good indicator evaluating the progression of the nutritional status. [10] In the population of heart failure, BW gain ≧ 0.91 kg over three days was a significant indicator for heart function decompensation, [13] while a BW loss ≧ 6% at any time during follow-up was a strong predictor of worse 8-months survival. [37] Similarly, a BW loss > 5% during follow-up within a 6-month period is a significant risk factor for death and CV death in patients post myocardial infarction. [12] As for the CS patients, a BW loss ≧ 10% in the six months before the admission was an association with a prolonged hospital LOS. [9]

In the current study, we found that the postoperative mortality was raised when the perioperative BW change increased to 2–15% (estimated 1.2–9 kg in a 60-kg adult) during a period with a mean value of 4.4 days and a median value of 3 days. Comparing with these previous studies, we demonstrated that the patient survival could be affected by a smaller BW change within a shorter observation period. Moreover, the scale of BW change in the current study was comparable with the findings in studies mentioned above when taking the length of follow-up period into consideration.

### Fluid status changes and patient outcomes

When discussing the BW change, fluid balance should be taken into consideration. [38] Among critically ill patients with AKI, a fluid accumulation of >10% BW was associated with a higher 30-days, 60-days, and in-hospital mortality in the western population. **[39]** Similar findings could also be seen in the Asian population. **[18]** A multicenter prospective study conducted by Wang et al. found that the fluid accumulation of >10% BW within the first three days of ICU admission was an independent risk factor for the occurrence of AKI and the increased severity of AKI. In these critically ill patients with AKI, the mean fluid balance within the first three days was 1.4 liters, which was higher in the non-survivors than in the survivors (2.77 versus 0.93 liters, p<0.001). Moreover, the cumulative fluid amount in the first three days was an independent predictor of 28-day mortality. **[18]** Furthermore, a meta-analysis enrolling 5095 patients from 12 cohort studies confirmed that fluid status, regarding either predefined “fluid overload” (categorical variables) or mean fluid balance (continuous variables), was positively associated with mortality in patients with AKI. **[17]**

As for the patients received CS, an accumulated fluid balance > 6.5 ml/kg BW (estimated 390 ml in a 60-kg adult) within the first 72 hrs postoperatively was associated with a longer LOS in those with oliguria, [40] and a fluid accumulation of ≧ 10% BW after CS during the ICU stay was associated with a higher mortality and a longer ICU LOS. [41] Whereas in the CS patients with RRT-requiring AKI, the non-survivors had significant higher fluid accumulation before RRT (8.8% versus 6.1%), after RRT (6.9% versus -0.5%), and during the whole ICU stay (18.0% versus 7.5%) than the survivors. Moreover, a fluid accumulation ≧ 7.2% during whole ICU stay is demonstrated as the cut-point for 90-day mortality. [42]

These three investigations in postoperative CS population [40–42] evaluated the fluid balance during the ICU stay, in which the period before and during the CS was not specified. While the current study evaluated the BW change at different period section including the preoperative, the perioperative, and the postoperative stages. Moreover, we found the perioperative BW change, which overwhelmed the BW changes at other period sections, showed a significant predictive value of mortality. Besides, the magnitude of the BW change in our study is significantly smaller than the fluid balance in other studies. The existence of the insensible fluid losses which vary depending on the environment and the disease process, [43] and the lean body weight loss secondary to the high catabolic status should take the main responsibility for the gap.

The current study echoed the concept that more intraoperative or perioperative fluid accumulation is associated with adverse postoperative prognoses, [44] and provided a simple and useful BW measurement to predict patient outcomes.

### Limitations

Several limitations of this study should be addressed. First, as an observational study, it is subject to bias. Second, current study enrolled a selected population of CS patients with RRT-requiring postoperative AKI. Thus the results may not serve as a representative sample of the all postoperative AKI patients, or AKI patients without RRT, throughout the world. Third, there is no optimal indicator to quantify the change of nutritional status, and the change of BW which is related to catabolic status, during the perioperative stage.

## Conclusions

Perioperative BW change was independently associated with an increased risk for 30-day postoperative mortality in CS patients with RRT-requiring AKI. These findings underscore the need for further studies using more optimal indicators to differentiate the effects of nutritional change and fluid retention.

## Supporting information

S1 Appendix Dataset of perioperative body weight change and outcomes. (XLSX)

S1 Table Complete clinical variables at hospital admission of the two groups. (DOC)

S2 Table Complete clinical variables at ICU admission of the two groups. (DOC)

S3 Table Complete clinical variables at renal replacement therapy initiation of the two groups. (DOC)

S4 Table Complete multivariate model for predicting 30-day mortality after the operation. (DOC)
