# Supplementary material for: Perioperative body weight change is associated with in-hospital mortality in cardiac surgical patients with postoperative acute kidney injury
Source: PLoS One. 2017 Nov 17;12(11):e0187280. doi: 10.1371/journal.pone.0187280 (PMC5693407; doi:10.1371/journal.pone.0187280)
Supplement: S1 Table — (DOC) [file pone.0187280.s002.doc]

**S1 Table. Complete clinical variables at hospital admission of the two groups.**

| **Variable** | **Survivors**  **(n=124)** | **Non- Survivors**  **(n=64)** | **p-value** |
| --- | --- | --- | --- |
| **Clinical parameters** | |  |  |
| BW, kg | 62.0 ± 12.6 | 58.5 ± 10.2 | 0.055 |
| BMI, kg/m^2^ | 23.7 ± 3.8 | 23.0 ± 3.5 | 0.247 |
| HR, /min | 83.4 ± 18.2 [80, 106] | 84.3 ± 15.5 [84, 72] | 0.358^*^ |
| RR, /min | 19.9 ± 4.2 [133.5, 149.0] | 20.4 ± 3.6 [128.0, 98.0] | 0.292^*^ |
| SBP, mmHg | 132.7 ± 26.7 [72, 94] | 125.9 ± 20.0 [72.5, 72] | 0.090^*^ |
| DBP, mmHg | 72.8 ± 15.0 | 72.6 ± 14.2 | 0.914 |
| MAP, mmHg | 92.8 ± 16.6 | 90.3 ± 14.4 | 0.323 |
| **Laboratory tests** |  |  |  |
| WBC, 10^3^/uL | 8.4 ± 3.9 [7.7, 2.6] | 8.2 ± 3.2 [7.8, 16.2] | 0.586^*^ |
| Hemoglobin, g/dL | 11.4 ± 2.5 [11.2, 14.5] | 12.2 ± 2.6 [12.2, 12.5] | 0.020^*^ |
| Hematocrit, % | 34.6 ± 7.6 [33.4, 44.1] | 37.1 ± 7.6 [36.3, 35.4] | 0.023^*^ |
| Platelet, 10^3^/uL | 212.6 ± 83.7 [201.5, 548.0] | 241.1 ± 298.6 [180.5, 2408.0] | 0.324^*^ |
| BUN, mg/dL | 46.7 ± 29.5 [41.2, 118.7] | 34.4 ± 34.4 [27.2, 89.7] | 0.010^*^ |
| Creatinine, mg/dL | 3.4 ± 6.1 [2.1, 65.4] | 1.9 ± 1.4 [1.4, 7.4] | 0.002^*^ |
| eGFR, ml/min/1.73m^2^ | 39.5 ± 29.5 [33.4, 145.6] | 51.5 ± 27.7 [50.2, 130.4] | 0.002^*^ |
| Albumin, g/dL | 3.8 ± 0.6 | 4.0 ± 0.6 | 0.130 |
| Sodium, mEq/L | 136.5 ± 4.9 [137, 25] | 136.3 ± 5.1 [137, 29] | 0.711^*^ |
| Potassium, mEq/L | 4.4 ± 0.8 [4.3, 3.5] | 4.4 ± 0.7 [4.2, 3.0] | 0.805^*^ |
| GOT, IU/L | 56.9 ± 120.8 [26, 1153] | 155.9 ± 665.5 [34, 5318] | 0.002^*^ |
| Bil-T, mg/dL | 1.1 ± 1.1 [0.7, 8.1] | 1.4 ± 1.3 [1.0, 7.2] | 0.008^*^ |

**Note:** Categorical variables were expressed as numbers (percentages) and analyzed using Chi-square test, or Fisher’s exact test if the expected value of any box is ≦5.

Continuous variables with normal distribution were expressed as “mean ± standard deviation” and analyzed using independent t-test.

^*^Continuous variables with non-normal distribution were expressed as “mean ± standard deviation [median, interquartile range]” and compared using Mann-Whitney U test.

**Abbreviations:** Bil-T, total bilirubin; BMI, body mass index; BUN, blood urea nitrogen; BW, body weight; DBP, diastolic blood pressure; eGFR, estimated glomerular filtration rate; GOT, glutamate oxaloacetate transaminase; HR, heart rate; MAP, mean arterial pressure; RR, respiratory rate; SBP, systolic blood pressure; WBC, white blood cell.
