# Supplementary material for: Perioperative body weight change is associated with in-hospital mortality in cardiac surgical patients with postoperative acute kidney injury
Source: PLoS One. 2017 Nov 17;12(11):e0187280. doi: 10.1371/journal.pone.0187280 (PMC5693407; doi:10.1371/journal.pone.0187280)
Supplement: S2 Table — (DOC) [file pone.0187280.s003.doc]

**S2 Table. Complete clinical variables at intensive care unit admission of the two groups.**

| **Variable** | **Survivors**  **(n=124)** | **Non- Survivors**  **(n=64)** | **p-value** |
| --- | --- | --- | --- |
| **Clinical parameters** |  |  |  |
| BW, kg | 61.8 ± 12.4 | 60.4 ± 10.2 | 0.457 |
| BMI, kg/m^2^ | 23.7 ± 4.0 | 23.8 ± 3.7 | 0.898 |
| HR, /min | 98.5 ± 21.2 | 106.6 ± 20.1 | 0.012 |
| RR, /min | 14.4 ± 4.5 | 14.2 ± 3.3 | 0.671 |
| SBP, mmHg | 125.2 ± 32.2 | 108.7 ± 26.6 | 0.001 |
| DBP, mmHg | 63.9 ± 16.5 | 58.5 ± 15.6 | 0.030 |
| MAP, mmHg | 84.3 ± 19.6 | 75.2 ± 16.9 | 0.002 |
| CVP, mmHg | 12.0 ± 5.3 | 10.4 ± 4.9 | 0.039 |
| IE, mcg/kg/min | 30.6 ± 243.7 [5.0, 2719.4] | 14.1 ± 26.3 [6.6, 192.9] | 0.403^*^ |
| Urine output, ml/day | 660.7 ± 692.5 | 781.1 ± 708.7 | 0.264 |
| **Laboratory tests** |  |  |  |
| WBC., 10^3^/uL | 11.7 ± 4.8 [11.4, 26.0] | 10.5 ± 5.7 [9.9, 3.4] | 0.020^*^ |
| Hemoglobin, g/dL | 11.4 ± 2.2 [11.1, 14.5] | 11.6 ± 2.0 [11.6, 7.5] | 0.570^*^ |
| Hematocrit, % | 34.8 ± 7.7 | 35.1 ± 5.8 | 0.803 |
| Platelet, 10^3^/uL | 167.7 ± 72.1 [158.5, 471.0] | 144.8 ± 63.9 [143.5, 334.2] | 0.066^*^ |
| BUN, mg/dL | 40.4 ± 21.4 [36.4, 118.2] | 33.3 ± 21.2 [28.1, 109.9] | 0.007^*^ |
| Creatinine, mg/dL | 3.3 ± 5.1 [2.4, 55.6] | 2.1 ± 1.3 [1.7, 7.2] | 0.003^*^ |
| eGFR, ml/min/1.73m^2^ | 32.9 ± 24.6 [26.1, 146.1] | 39.4 ± 16.1 [41.2, 101.6] | 0.002^*^ |
| Albumin, g/dL | 3.4 ± 0.6 | 3.0 ± 0.7 | 0.004 |
| Sodium, mEq/L | 139.0 ±7.3 [139.0, 32.0] | 144.2 ± 7.8 [143.0, 48.0] | <0.001^*^ |
| Potassium, mEq/L | 4.2 ± 0.8 | 4.2 ± 0.7 | 0.977 |
| Calcium, mg/dL | 1.2 ± 0.1 [1.2, 0.8] | 1.1 ± 0.2 [1.1, 1.4] | 0.082^*^ |
| GOT, IU/L | 120.5 ± 155.7 [66.5, 1210.0] | 318.2 ± 442.0 [167.5, 2084.0] | <0.001^*^ |
| Bil-T, mg/dL | 2.9 ± 9.4 [1.3, 102.0] | 2.9 ± 2.6 [2.0, 14.0] | <0.001^*^ |
| Sugar, mg/dL | 223.5 ± 91.8 | 228.9 ± 79.3 | 0.691 |
| Lactate, mEq/L | 6.4 ± 5.7 [4.7, 24.6] | 9.1 ± 6.5 [7.0, 23.8] | 0.002^*^ |
| PH | 7.4 ± 0.1 [7.4, 0.9] | 7.4 ± 0.1 [7.4, 0.6] | 0.950^*^ |
| PaCO2, mmHg | 34.9 ± 9.3 [33.3, 54.7] | 33.7 ± 9.6 [31.9, 49.3] | 0.276^*^ |
| PaO2, mmHg | 140.8 ± 79.8 [113.5, 427.5] | 167.5 ± 87.0 [133.4, 346.3] | 0.017^*^ |
| HCO3, mEq/L | 20.8 ± 3.576 | 20.4 ± 4.5 | 0.554 |
| **Severity scores** |  |  |  |
| GCS, points^#^ | 13.1 ± 4.1 [15, 12] | 10.3 ± 5.2 [15, 12] | <0.001^*^ |
| APACHE-II, points | 11.6 ± 6.9 [10.0, 31.0] | 12.3 ± 7.1 [10.0, 26.0] | 0.506^*^ |
| SOFA Score, points | 9.0 ± 3.2 [9, 15] | 11.1 ± 3.2 [11, 15] | <0.001^*^ |
| LODS, points | 14.2 ± 3.4 [15, 12] | 16.5 ± 1.2 [17, 5] | <0.001^*^ |
| MODS, points | 6.6 ± 3.1 | 7.6 ± 3.1 | 0.053 |

**Note:** Continuous variables with normal distribution were expressed as “mean ± standard deviation” and analyzed using independent t-test.

^*^Continuous variables with non-normal distribution were expressed as “mean ± standard deviation [median, interquartile range]” and compared using Mann-Whitney U test.

**Abbreviations:** APACHE, Acute Physiology and Chronic Health Evaluation; Bil-T, total bilirubin; BMI, body mass index; BUN, blood urea nitrogen; BW, body weight; CVP, central venous pressure; DBP, diastolic blood pressure; eGFR, estimated glomerular filtration rate; GCS, Glasgow Coma Scale; GOT, glutamate oxaloacetate transaminase; HCO3, bicarbonate; HR, heart rate; IE, inotropic equivalents; LODS, Logistic Organ Dysfunction Score; MAP, mean arterial pressure; MODS, Multiple Organ Dysfunction Score; PaCO2, partial arterial pressure of carbon dioxide; PaO2, partial arterial pressure of oxygen; PH, acidity; RR, respiratory rate; SBP, systolic blood pressure; SOFA, Sequential Organ Failure Assessment; WBC, white blood cell.
