# Supplementary material for: Perioperative body weight change is associated with in-hospital mortality in cardiac surgical patients with postoperative acute kidney injury
Source: PLoS One. 2017 Nov 17;12(11):e0187280. doi: 10.1371/journal.pone.0187280 (PMC5693407; doi:10.1371/journal.pone.0187280)
Supplement: S3 Table — (DOC) [file pone.0187280.s004.doc]

**S3 Table. Complete clinical variables at renal replacement therapy initiation of the two groups.**

| **Variable** | **Survivors**  **(n=124)** | **Non- Survivors**  **(n=64)** | **p-value** |
| --- | --- | --- | --- |
| **Clinical parameters** |  |  |  |
| BW, kg | 64.1 ± 13.1 | 62.1 ± 11.3 | 0.316 |
| BMI, kg/m^2^ | 24.5 ± 3.9 | 24.4 ± 4.2 | 0.882 |
| HR, /min | 99.1 ± 20.2 | 109.1 ± 21.1 | 0.002 |
| RR, /min | 15.9 ± 5.0 [15, 25] | 15.7 ± 5.1 [16, 23] | 0.803^*^ |
| SBP, mmHg | 123.5 ± 27.4 | 105.8 ± 24.9 | <0.001 |
| DBP, mmHg | 63.1 ± 13.9 | 57.5 ± 15.1 | 0.011 |
| MAP, mmHg | 83.3 ± 16.2 | 73.6 ± 16.2 | <0.001 |
| CVP, mmHg | 12.3 ± 5.1 [12, 25] | 14.1 ± 5.4 [13, 31] | 0.013^*^ |
| IE, mcg/kg/min | 34.8 ± 243.5 [6.7, 2719.4] | 25.2 ± 24.3 [19.1, 168.2] | <0.001^*^ |
| Urine output, ml/day | 442.1 ± 410.8 [330.0, 1730.0] | 415.4 ± 474.1 [297.5, 2619.0] | 0.448^*^ |
| **Laboratory tests** |  |  |  |
| WBC 10^3^/uL | 13. 3 ± 5.6 [12.7, 3.5] | 12.1 ± 6.3 [11.2, 3.4] | 0.101^*^ |
| Hemoglobin, g/dL | 12.1 ± 7.4 [11.2, 83.7] | 12.6 ± 8.5 [11.5, 70.0] | 0.660^*^ |
| Hematocrit, % | 34.2 ± 6.3 | 34.8 ± 5.8 | 0.567 |
| Platelet, 10^3^/uL | 133.3 ± 62.3 [129.5, 344.0] | 108.4 ± 64.6 [94.5, 350.0] | 0.004^*^ |
| BUN, mg/dL | 54.0 ± 29.0 [49.9, 156.7] | 45.1 ± 24.8 [36.9, 99.4] | 0.029^*^ |
| Creatinine, mg/dL | 4.3 ± 6.7 [3.2, 73.4] | 2.7 ± 1.6 [2.1, 7.0] | <0.001^*^ |
| eGFR, ml/min/1.73m^2^ | 24.0 ± 17.3 [19.3, 90.8] | 30.4 ± 14.6 [29.6, 61.0] | 0.001^*^ |
| Albumin, g/dL | 3.3 ± 0.6 | 3.0 ± 0.7 | 0.017 |
| Sodium, mEq/L | 140.3 ± 6.6 [139.3, 36.2] | 145.9 ± 7.5 [145.0, 37.0] | <0.001^*^ |
| Potassium, mEq/L | 4.5 ± 0.8 | 4.3 ± 0.8 | 0.245 |
| Calcium, mg/dL | 1.2 ± 0.1 [1.2, 0.9] | 1.1 ± 0.1 [1.1, 0.7] | 0.006^*^ |
| GOT, IU/L | 208.9 ± 516.6 [69.0, 5067.0] | 649.3 ± 1706.8 [223.0, 12999.0] | <0.001^*^ |
| Bil(T), mg/dL | 2.4 ± 3.2 [1.3, 23.6] | 3.8 ± 4.4 [2.3, 30.2] | <0.001^*^ |
| Sugar, mg/dL | 220.8 ± 120.3 [192.0, 772.8] | 196.0 ± 70.1 [199.5, 318.0] | 0.538^*^ |
| Lactate, mEq/L | 5.4 ± 5.4 [3.2, 26.5] | 8.6 ± 6.4 [5.9, 26.1] | <0.001^*^ |
| PH | 7.4 ± 0.1 [7.4, 0.9] | 7.4 ± 0.1 [7.4, 0.5] | 0.596^*^ |
| PaCO2, mmHg | 31.8 ± 8.4 [30.9, 56.5] | 31.8 ± 9.5 [29.7, 66.3] | 0.826^*^ |
| PaO2, mmHg | 137.8 ± 70.4 [118.7, 410.7] | 147.6 ± 81.3 [118.2, 456.8] | 0.532^*^ |
| HCO3, mEq/L | 20.8 ± 4.3 | 20.1 ± 4.2 | 0.267 |
| **Severity scores** |  |  |  |
| GCS, points^#^ | 13.4 ± 3.7 [15, 12] | 9.4 ± 5.1 [8, 12] | <0.001^*^ |
| APACHE-II, points | 10.7 ± 5.8 [9.5, 31.0] | 14.6 ± 7.3 [16.0, 29.0] | <0.001^*^ |
| SOFA Score, points | 10.2 ± 3.1 [10.0, 13.0] | 13.9 ± 3.4 [14.0, 14.0] | <0.001^*^ |
| LODS, points | 13.6 ± 3.5 [15.0, 13.0] | 15.8 ± 1.7 [17.0, 5.0] | <0.001^*^ |
| MODS, points | 7.1 ± 3.0 [6.5, 16.0] | 10.0 ± 3.6 [9.5, 17.0] | <0.001^*^ |

**Note:** Continuous variables with normal distribution were expressed as “mean ± standard deviation” and analyzed using independent t-test.

^*^Continuous variables with non-normal distribution were expressed as “mean ± standard deviation [median, interquartile range]” and compared using Mann-Whitney U test.

**Abbreviations:** APACHE, Acute Physiology and Chronic Health Evaluation; Bil-T, total bilirubin; BMI, body mass index; BUN, blood urea nitrogen; BW, body weight; CVP, central venous pressure; DBP, diastolic blood pressure; eGFR, estimated glomerular filtration rate; GCS, Glasgow Coma Scale; GOT, glutamate oxaloacetate transaminase; HCO3, bicarbonate; HR, heart rate; IE, inotropic equivalents; LODS, Logistic Organ Dysfunction Score; MAP, mean arterial pressure; MODS, Multiple Organ Dysfunction Score; PaCO2, partial arterial pressure of carbon dioxide; PaO2, partial arterial pressure of oxygen; PH, acidity; RR, respiratory rate; SBP, systolic blood pressure; SOFA, Sequential Organ Failure Assessment; WBC, white blood cell.
