# Supplementary material for: Perioperative body weight change is associated with in-hospital mortality in cardiac surgical patients with postoperative acute kidney injury
Source: PLoS One. 2017 Nov 17;12(11):e0187280. doi: 10.1371/journal.pone.0187280 (PMC5693407; doi:10.1371/journal.pone.0187280)
Supplement: S4 Table — (DOC) [file pone.0187280.s005.doc]

**S4 Table. Complete multivariate model for predicting 30-day mortality after the operation.**

| Step |  | **B** | **SE** | **HR** | **95 % CI** | **p-value** |
| --- | --- | --- | --- | --- | --- | --- |
| 1 | Peri-op BW change ^a^ | 0.08 | 0.02 | 1.06 | 1.04-1.13 | <0.001 |
|  | Post-RRT BW change ^a^ | 0.06 | 0.01 | 1.06 | 1.03-1.08 | <0.001 |
|  | DM ^b^ | 0.51 | 0.35 | 1.66 | 0.84-3.28 | 0.142 |
|  | CKD ^b^ | -1.90 | 1.03 | 0.15 | 0.02-1.12 | 0.065 |
|  | eGFR at admission ^a^ | 0.00 | 0.01 | 1.00 | 0.99-1.01 | 0.441 |
|  | Hemoglobin at admission ^a^ | 0.09 | 0.07 | 1.09 | 0.96-1.24 | 0.192 |
|  | Bil-T at admission ^a^ | -0.03 | 0.10 | 0.97 | 0.80-1.18 | 0.770 |
|  | GOT at admission ^a^ | 0.00 | 0.00 | 1.00 | 1.00-1.00 | 0.849 |
|  | Peri-op PRBC transfusion ^a^ | -0.25 | 0.12 | 0.78 | 0.62-0.98 | 0.032 |
|  | Peri-op FFP transfusion ^a^ | 0.05 | 0.04 | 1.05 | 0.98-1.14 | 0.186 |
|  | IABP ^b^ | 0.45 | 0.35 | 1.57 | 0.79-3.14 | 0.202 |
| 2 | Peri-op BW change ^a^ | 0.09 | 0.04 | 1.09 | 1.00-1.19 | 0.045 |
|  | Post-RRT BW change ^a^ | 0.08 | 0.03 | 1.08 | 1.03-1.14 | 0.002 |
|  | Peri-op PRBC transfusion ^a^ | -0.14 | 0.16 | 0.87 | 0.64-1.19 | 0.396 |
|  | CAVH (with ECMO) ^b^ | 1.93 | 0.71 | 6.91 | 1.73-27.70 | 0.006 |
|  | Post-op albumin ^a^ | 0.80 | 0.55 | 2.23 | 0.75-6.58 | 0.148 |
|  | Post-op HR ^a^ | 0.04 | 0.01 | 1.04 | 1.01-1.07 | 0.004 |
|  | Post-op MAP ^a^ | -0.03 | 0.02 | 0.97 | 0.94-1.01 | 0.105 |
|  | Post-op CVP ^a^ | 0.00 | 0.04 | 1.00 | 0.91-1.09 | 0.926 |
|  | Post-op GCS ^a^ | -0.14 | 0.07 | 0.87 | 0.76-0.99 | 0.037 |
|  | Post-op PaO2 ^a^ | 0.00 | 0.00 | 1.00 | 0.99-1.00 | 0.181 |
|  | Post-op Bil-T ^a^ | -0.02 | 0.04 | 0.98 | 0.92-1.05 | 0.65 |
|  | Post-op eGFR ^a^ | -0.01 | 0.02 | 0.99 | 0.96-1.02 | 0.538 |
|  | Post-op Sodium ^a^ | 0.02 | 0.04 | 1.02 | 0.95-1.09 | 0.553 |
|  | Post-op Lactate ^a^ | -0.05 | 0.05 | 0.95 | 0.86-1.06 | 0.37 |
|  | Post-op GOT ^a^ | 0.00 | 0.00 | 1.00 | 1.00-1.00 | 0.004 |
|  | Post-op WBC ^a^ | 0.00 | 0.00 | 1.00 | 1.00-1.00 | 0.031 |
|  | Post-op SOFA Score ^a^ | 0.06 | 0.09 | 1.06 | 0.89-1.27 | 0.515 |
|  | Post-op LODS ^a^ | 0.83 | 0.34 | 2.30 | 1.18-4.46 | 0.014 |
| 3 | Peri-op BW change ^a^ | 0.09 | 0.02 | 1.10 | 1.05-1.15 | <0.001 |
|  | Post-RRT BW change ^a^ | 0.07 | 0.01 | 1.07 | 1.05-1.10 | <0.001 |
|  | Peri-op PRBC transfusion ^a^ | -0.30 | 0.12 | 0.74 | 0.59-0.94 | 0.013 |
|  | CAVH (with ECMO) ^b^ | 1.32 | 0.46 | 3.74 | 1.53 -9.19 | 0.004 |
|  | Post-op HR ^a^ | 0.02 | 0.01 | 1.02 | 1.00 -1.03 | 0.073 |
|  | Post-op GCS ^a^ | -0.07 | 0.06 | 0.93 | 0.83-1.05 | 0.267 |
|  | Post-op LODS ^a^ | 0.10 | 0.13 | 1.11 | 0.85-1.44 | 0.455 |
|  | HR at RRT ^a^ | 0.02 | 0.01 | 1.02 | 1.00-1.04 | 0.019 |
|  | MAP at RRT ^a^ | -0.03 | 0.01 | 0.97 | 0.95-0.99 | 0.011 |
|  | CVP at RRT ^a^ | 0.04 | 0.04 | 1.05 | 0.97-1.13 | 0.272 |
|  | GCS at RRT ^a^ | -0.06 | 0.09 | 0.95 | 0.79-1.14 | 0.551 |
|  | Bil-T at RRT ^a^ | -0.04 | 0.04 | 0.96 | 0.89-1.04 | 0.288 |
|  | eGFR at RRT ^a^ | 0.00 | 0.02 | 1.00 | 0.97-1.03 | 0.904 |
|  | Sodium at RRT ^a^ | 0.03 | 0.02 | 1.03 | 0.99-1.08 | 0.186 |
|  | Lactate at RRT ^a^ | -0.03 | 0.03 | 0.97 | 0.91-1.04 | 0.370 |
|  | GOT at RRT ^a^ | 0.00 | 0.00 | 1.00 | 1.00-1.00 | 0.634 |
|  | Calcium at RRT ^a^ | -1.35 | 1.26 | 0.26 | 0.02-3.04 | 0.282 |
|  | Platelet at RRT ^a^ | 0.00 | 0.00 | 1.00 | 1.00-1.01 | 0.481 |
|  | IE at RRT ^a^ | -0.01 | 0.01 | 0.99 | 0.98-1.00 | 0.121 |
|  | APACHE-II at RRT ^a^ | -0.05 | 0.06 | 0.95 | 0.85-1.07 | 0.383 |
|  | SOFA Score at RRT ^a^ | 0.25 | 0.10 | 1.28 | 1.05-1.56 | 0.016 |
|  | LODS at RRT ^a^ | 0.20 | 0.14 | 1.22 | 0.93-1.59 | 0.146 |
|  | MODS at RRT ^a^ | -0.03 | 0.13 | 0.97 | 0.75-1.25 | 0.822 |
| 4 | Peri-op BW change ^a^ | 0.07 | 0.02 | 1.07 | 1.03-1.12 | 0.001 |
|  | Post-RRT BW change ^a^ | 0.06 | 0.01 | 1.06 | 1.04-1.08 | <0.001 |
|  | Peri-op PRBC transfusion ^a^ | -0.43 | 0.12 | 0.65 | 0.51-0.82 | <0.001 |
|  | CAVH (with ECMO) ^b^ | 1.41 | 0.41 | 4.09 | 1.85-9.05 | 0.001 |
|  | HR at RRT ^a^ | 0.02 | 0.01 | 1.02 | 1.01-1.03 | 0.001 |
|  | MAP at RRT^a^ | -0.02 | 0.01 | 0.98 | 0.97-1 | 0.024 |
|  | SOFA Score at RRT ^a^ | 0.20 | 0.04 | 1.22 | 1.13-1.33 | <0.001 |
|  |  |  |  |  |  |  |
| 5 | Peri-op BW change ^a^ | 0.06 | 0.02 | 1.07 | 1.02-1.11 | 0.002 |
|  | Post-RRT BW change ^a^ | 0.06 | 0.01 | 1.06 | 1.03-1.08 | <0.001 |
|  | Peri-op PRBC transfusion ^a^ | -0.39 | 0.12 | 0.68 | 0.54-0.85 | 0.001 |
|  | CAVH (with ECMO) ^b^ | 1.35 | 0.41 | 3.87 | 1.74-8.61 | 0.001 |
|  | HR at RRT ^a^ | 0.02 | 0.01 | 1.02 | 1.01-1.03 | 0.001 |
|  | MAP at RRT^a^ | -0.02 | 0.01 | 0.98 | 0.97-1 | 0.037 |
|  | SOFA Score at RRT ^a^ | 0.21 | 0.04 | 1.23 | 1.13-1.33 | <0.001 |
|  |  | 1.94 | 0.95 | 6.94 | 1.09-44.38 | 0.041 |

**Note:** The analysis was performed using stepwise selection method of multivariate Cox proportional hazards model. Variables were selected to put into multivariate analysis if they had a p ≦ 0.05 on univariate analysis. These selected variables for multivariate analysis included DM, CKD, and some laboratory tests (hemoglobin, eGFR, GOT, and Bil-T) at hospital admission; the clinical variables (HR, MAP, CVP, IABP, perioperative PRBC and FFP transfusion, perioperative BW change and BW change after RRT), the laboratory tests (WBC, eGFR, sodium, GOT, Bil-T, albumin, lactate, PaO2), and the severity scores (GCS, SOFA Score and LODS) at postoperative stage; as well as the clinical variables [CAVH(with ECMO), HR, MAP, CVP, IE], the laboratory tests (platelet, eGFR, sodium, calcium, GOT, albumin, Bil-T, lactate) and the severity scores (GCS, APACHE-II, SOFA Score, LODS and MODS) at RRT initiation. Also, a propensity score composed of age, body mass index, gender, CCI, eGFR, as well as the days between hospital admission to surgery, was applied for the baseline difference adjustment in the multivariate Cox proportional hazards model.

Duration for analysis is measured using calendar days from cardiac surgery to mortality.

^a^ every increment of 1 unit or point; ^b^ with versus without; “at RRT” denotes “at the timing of RRT initiation.”

**Abbreviations:** APACHE, Acute Physiology and Chronic Health Evaluation; B, beta coefficient; Bil-T, total bilirubin; BW, body weight; CAVH, continuous arteriovenous hemofiltration; CI, confidence interval; CKD, chronic kidney disease; CVP, central venous pressure; DM, diabetes mellitus; ECMO, extracorporeal membrane oxygenation; eGFR, estimated glomerular filtration rate; GOT, glutamate oxaloacetate transaminase; FFP, fresh frozen plasma; GCS, Glasgow Coma Scale; HR, heart rate; HR, hazard ratio; IABP, Intra-aortic balloon pump; IE, inotropic equivalent; LODS, Logistic Organ Dysfunction Score; MAP, mean arterial pressure; MODS, Multiple Organ Dysfunction Score; PaO2, partial arterial pressure of oxygen; Peri-op, perioperative; Post-op, postoperative; PRBC, packed red blood cells; RRT, renal replacement therapy; SE, standard error; SOFA, Sequential Organ Failure Assessment; WBC, white blood cell.
